# Case of Diseased Liver Cured by Strong Mercurial Excitement

**Published:** 1814-06

**Authors:** Robert Semple

**Affiliations:** Member of the Royal College of Surgeons in London, &c. 85, High-street, Islington


*For the Medical and Physical Journal.*

### *Case of Diseased Liver cured by strong Mercurial Excitement; by* Mr. R. Semple.

JOHN CRAWLEY, aged 40, a labourer, applied to me as a parish pauper for relief for a pain in his thigh and knee, which he said came on from cold. There was nothing to be seen, and it was treated as a rheumatic affection, but without relief. The outside of the knee being the part chiefly affected, he was blistered and cupped, which gave him a little ease. Thinking he had no chance for a cure as an out-patient, I recommended his removal into the work-house of this parish. An abstract of his treatment there is given as follows from my case book.

This man's case, which was a severe affection of the liver, put on so alarming an appearance during the progress of the disease, that I had no hopes of his recovery. Indeed my belief that this patient would die continued till the mercury began to have a very evident effect on his system. This then is another proof that a disease may go on, and continue unabated, till the action of the mercury on the constitution becomes apparent. To obviate constipation, purgatives of various kinds were administered to him, with relief of symptoms.

Feb. 21, 1814.—Complains of much pain in the region of the bladder. Makes water very frequently, and in small quantity. Hab. Calomel, gr. ij. m. & v.

23d.—Pain in the region of the bladder still severe. Applic. Emplastr. Lyttæ parti dolenti. Cont. Pil. Calomel.

26th.—The blister rose well, and afforded relief. Makes water more freely. Bowels costive. Omitt. Pil. Calom. Hab. Sulph. Sodæ, ℥i. statim.

27th.

27th.—Physic did not operate.

℞. Pulv. Jalap, ʒſs. s. sumend.

28th.—Physic operated. Feels better. Complains of pains in the loins, and about the kidneys. Hab. Pilul. Hydrarg. No. ij. omni nocte.

March 2d.—As last report. Cont. Pil. Hydrarg.

3d.—Feels better. One motion. Blister healed. Makes water more freely. Hab. Cerevis. Londin. ℔j. in die.

4th.—A hæmorrhoidal tumor gives much pain. Costive.

℞. Pulv. Rhei. ℈j. s. s.

6th.—Says his urine is voided by drops, but at healthy intervals. Cont. Pil. Hydr.

8th.—Complains of much pain in the inferior edge of the liver, especially on pressure. Bowels open. Cont. Pilul. Hydr.

12th.—Pain still continues in the inferior edge of the liver. Stools greenish. Cont. Pilul.

13th.—One motion. Pil. Opii, gr. j. h. s.

14th.—Pain abated by the opium, but it has again come on. Mouth not affected. Omitt. Pil. Hydrarg. Perfric. Ung. Hydrarg. fort. ʒj. Region. Hepat. o. n.

15th.—Had a loose stool, mixed with bile. Cont. Ung.

17th.—Finds relief from the friction. Cont. Unguent.

20th.—Complains again of the pain. Pil. Opii, gr. iſs. h. s. Cont. Unguent. Hydrarg.

21st.—Rep. Ung. Hydr. & Opium, n. h.

24th.—No change. Rep. Ung. Augend. dos. Opii. ad gr. ij.

26th.—The region of the liver is so tender that he can no longer bear the friction. Perfric. Ung. Hyd. ʒi. femorib. B. costive.

℞. Submur. Hydrarg. gr. iv.
Pulv. Jalap. gr. x.
——— Zingib. gr. vi. M. ft. pulv. s. s.

30th.—No abatement of the pain, which is somewhat lower than before. Applic. Empl. Lyttæ Ampl. parti dolenti Mitte Cerat. Sabin. parti vesicat. Cont. Ung. Hydrarg.

31st.—Blister rose well, but did not afford much relief. Rep. Ung. Hydr.

April 1.—Mouth not yet affected. Omitt. Ung. Hydrarg.

℞. Submur. Hydrarg. gr. xviij.
Mic. panis, q. s. ut. ft. pil. vj. Capt. unam. m. & v.

3d.—This morning he has vomited a considerable quantity of purulent looking matter, which has afforded much relief. Pain in the hepatic region abated. Mouth slightly affected. Cont. Medicamenta.

4th.

4th.—Brought up a little more of the matter mentioned yesterday. Cont. Pil. Calom. & Cerat. Sabin.

5th.—Appears to sink. On examining his back, I perceived a jutting out of three of the last dorsal vertebræ, and, on inquiry, he said he had received a sprain three years ago, in lifting a load. The spinous processes are very painful on slight pressure. No apparent inflammation. I suppose a dislocation has taken place at the period above-mentioned. Mouth sore, with considerable ptyalism. Omitt. Calomel. Pil. Opii, gr. ij. h. s. (I mean always the crude opium.)

6th.—Feels the pain still severe in the hepatic and umbilical regions, and says it is much greater than the blistered part. Curet. pars vesic. Cerat. Lap. Calam. Hab. Vin. Rubr. ℥vi. vice Cerevis. indies.

℞. Opii crud. gr. ij.

Mic. pan. q. s. ut fiant pil. iv. sumat 1m. omni horâ.

7th.—Has expectorated a considerable quantity of blood. Skin hot and moist. Pills eased the pain. Blister runs much. Hab. pilul. ut heri prescript. 2da quaque horâ.

8th.—Feels rather better. Pills seem to have a good effect. Cont. Pilul. ut heri.

9th.—This morning, on entering the Infirmary, I found this man asleep, which to the bystanders appeared the sleep of death. On touching him he awoke, and was covered with a cold clammy sweat. Pills afford relief. P. small and feeble, about 80. Omitt. Opium. Hab. Pulv. Cinch. palid. ʒſs. ex vini cyatho ter in die.

10th.—Says the bark disagrees with him. Omitt. Pulv. Cinch. Cont. Vinum.

15th.—Appears better. Cont. Vinum.

16th.—Mouth very sore. Feels and appears amazingly better. This morning my opinion of the issue of this case was changed, and I now began to entertain hopes of a speedy recovery. He had regained his appetite, and his countenance had the appearance of a convalescent. The action of the mercury on his system was now very great; and to this cause I attribute the very wonderful change which has taken place. To obviate constipation, I ordered him ʒiv. Sulph. Sodæ.

21st.—This man gains flesh and strength. Has no pain. His back gives him little uneasiness. Appetite pretty good. His convalescence now goes on very rapidly. He sits up in the bed through the day, and takes his food with a relish.

The rapidity of this man's recovery from a disease which put on so alarming an appearance, induces me to publish the case. Perhaps changing the mercurials occasionally was of advantage. Indeed I at one time almost despaired of ever affecting my patient's constitution. His symptoms were growing gradually worse, till the system became charged with the mercury. This case I consider important, as it leads us to a perseverance in the exhibition of that powerful and useful mineral, till the system becomes ultimately affected. The subject of mercurial action is ably discussed in your Number for December last, by Mr. D. H. Davies. I received much instruction from the perusal of his remarks. They are practical, and highly interesting.

ROBERT SEMPLE,

*Member of the Royal College of Surgeons in London, &c.*

85, *High-street, Islington,*

*April* 21, 1814.

